# Biological properties of a novel solution based on silver nanoclusters for arresting dentin caries

**DOI:** 10.3389/froh.2024.1408181

**Published:** 2024-07-12

**Authors:** Gustavo Fabián Molina, María Belén Cabalén, Juan Pablo Aranguren, Gustavo Ariel Pino, Michael Francis Burrow

**Affiliations:** ^1^ Facultad de Ciencias de la Salud, Universidad Católica de Córdoba, Córdoba, Argentina; ^2^ Centro Láser de Ciencias Moleculares, Universidad Nacional de Córdoba, Haya de la Torre s/n, Pabellón Argentina, Ciudad Universitaria, Córdoba, Argentina; ^3^ The Faculty of Dentistry, University of Hong Kong, Hong Kong, Hong Kong SAR China; ^4^ Becaria CONICET, Facultad de Ciencias de la Salud, Universidad Católica de Córdoba, Córdoba, Argentina; ^5^ INFIQC: Instituto de Investigaciones en Fisicoquímica de Córdoba (CONICET – UNC), Universidad Nacional de Córdoba, Haya de la Torre s/n, Pabellón Argentina, Ciudad Universitaria, Córdoba, Argentina; ^6^ Departamento de Fisicoquímicas, Facultad de Ciencias Químicas, Universidad Nacional de Córdoba, Haya de la Torre s/n, Pabellón Argentina, Ciudad Universitaria, Córdoba, Argentina

**Keywords:** silver diammine fluoride, dental caries, minimally invasive dentistry, nonrestorative caries treatment, nanotechnology

## Abstract

**Objectives:**

To test the biological properties of a novel non-restorative treatment method for arresting dentin caries based on silver nanoclusters (AgNCls) synthesized in polymethacrylic acid (PMAA).

**Methods:**

Synthesis of AgNCls was performed by photoreduction of AgNO_3_ in PMAA with 355 nm/wavelength light. AgNCls/PMAA was characterized by absorption/fluorescence spectroscopy and optical and atomic force microscopy. The stability of the clusters in an aerated PMAA solution was evaluated by means of fluorescence spectroscopy. Cytotoxicity was assessed using the MTT assay and antibacterial effect was determined for minimum inhibitory concentration (MIC), minimum bactericidal concentration (MBC) and colony forming (CFU) of *Streptococcus mutans* (*S. mutans*) and *Lactobacillus acidophilus* (*L. acidophilus*). 38% Silver Diammine Fluoride (SDF) was used for the control groups.

**Results:**

Chemical and structural identity of the clusters did not change within 9 months; Cell viability of 92%–89% was found after 24–48 h respectively. MIC and MBC were determined from 1:16 and 1:8 dilutions, respectively. Log CFU counts of *S. mutans,* and *L. acidophilus* treated with AgNCls/PMAA (3.4 ppm of silver) were significantly lower than in the control groups and even lower than when the same bacterial strains were treated with SDF (15,525 ppm of silver).

**Conclusions:**

AgNCls/PMAA presented chemical stability, acceptable cytotoxicity, and a potential antibacterial effect for strains associated with caries lesions at very low concentrations of silver.

## 1 Introduction

A non-restorative approach for arresting caries lesions is a relevant topic to Minimally Invasive Dentistry as this strategy is the most conservative for management. There is an array of products that have demonstrated promising results to recover demineralized enamel but fail to stop the progression of a lesion that has already reached the dentin. Silver Diammine Fluoride (38% SDF) has shown to be highly effective to arrest dentin caries due to its antibacterial effect of silver compounds (1) and the fluoride remineralizing properties (2, 3).

Even though SDF has proved to be an effective anti-caries agent, it is associated with certain side effects like gingival irritation, a metallic taste, and irreversible dark stains on the treated surfaces. Darkening of the arrested lesion is one of the main concerns when aesthetic restorations are sought. To avoid staining, different strategies have been proposed such as reducing silver oxidation by applying potassium iodide after SDF and/or masking these dark stains with the coverage of a restorative material such as glass ionomer cement or resin composite. Although there is an improvement in the appearance, aesthetic outcomes are still below expectations.

Traditional silver compounds cause tooth discolouration as a consequence of the oxidation of silver ions, depending on the amount applied and the frequency of application. To overcome these side effects, researchers have introduced Nano Silver Fluoride (NSF) and silver nanoparticles for caries management (1). NSF was shown to perform like SDF without tooth staining and has comparable preventive and antibacterial activities as SDF, claiming to be simple to use, economic and safe in children and adults (4). The use of silver nanoparticles (AgNPs) to replace the traditional stable solutions of AgNO_3_ has been shown to overcome the problem of silver oxidation (5). Moreover, the antibacterial properties of silver are preserved in its nanosized version even at very low concentration (6).

In line with these findings, a novel solution to arrest dentin caries lesions based on silver nanoclusters (AgNCls) synthesized in polymethacrylic acid (PMAA) has been reported to significantly harden simulated dentin caries lesions without staining or darkening the treated surface (7). In addition, the pre-treatment of these surfaces with AgNCls/PMAA increased the shear bond strength (SBS) of a restorative glass ionomer cement (GIC) which could eventually contribute to synergestic properties in a non-restorative/restorative system (8).

The aim of the present study was to assess biological properties of AgNCls/PMAA in order to determine its cytotoxicity, antibacterial effect and chemical stability to further characterize this development as a potential resource for the management of caries lesions.

## 2 Materials and methods

All experimental protocols were approved by the secretary of research and development, Universidad Católica de Cordoba, Argentina (SI-UCC research grants) and by the National Agency for Research under the research grant FONCYT-PICT2020 Serie A #00539. All methods were carried out in accordance with relevant guidelines and regulations.

### 2.1 Synthesis and characterization of AgNCls/PMAA

The synthesis of AgNCls/PMAA was carried out at room temperature by photoreduction of AgNO_3._

in the presence of PMAA with a lamp emitting at 355 nm, as reported in the literature (9–16). Aqueous solutions (distilled water of Milli-Q grade) of poly(methacrylic acid, sodium salt) in aqueous solution 30% wt. (Sigma Aldrich, M_w_ = 9500) and AgNO_3_ (Sigma-Aldrich, >99.8%) were mixed to obtain a final concentration of Ag^+^ (5 × 10^−4^ M) PMAA polymer in a ratio 5:1 (Ag^+^: MAA), which corresponds to five Ag^+^ ion per MAA unit. In all cases, the optimal pH conditions were constantly evaluated in the range of pH 5.5–6.5. The mixture was prepared in a flask, which was completely sealed by a Teflon septum. The flask was exposed to 355 nm light for 1 h, and then, the flask was covered with aluminum foil and kept at 4°C.

This composition was chosen, since its penetration into the demineralised dentin, its color stability on treated surfaces and the induced changes in hardness of the dentin surface, were already determined and reported in previous laboratory studies (7, 8). Consequently, this composition was chosen as a reference to compare with commercial 38% SDF (FAgamin—batch #8252-08/24, Tedequim, Córdoba, Argentina).

The total concentration of silver in this composite material is 54 ppm, while the total concentration of silver in 38% SDF is 248,400 ppm. Therefore, hereafter we will refer to the total silver concentration of both materials.

The AgNCls/PMAA characterization was performed using:

*Absorption spectroscopy:* The absorption spectra of the AgNCls/PMAA solution were measured with an UV-Vis spectrophotometer (UV-Vis 1800, Shimadzu) at different times after the synthesis (0 h to 9 months). All the spectra were taken against water as a blank.

*Fluorescence Spectroscopy:* The fluorescence of the AgNCls/PMAA solution was characterized using Fluorimeter (Nanolog, Horiba) by recording the Excitation-Emission-Matrix (EMM) at different times after the synthesis (0 h–9 months).

*Optical Microscopy:* Films of AgNCls/PMAA were analyzed via an optical microscope (Jobin-Yvon, Horiba) using 50× and 100× objectives. The films were prepared by drop-coating of AgNCls/PMAA solution. Before drop-coating, the borosilicate glass substrates (22 mm × 22 mm × 0.19 mm) were cleaned gently with deionised water and absolute ethanol and dried with a nitrogen flow. A volume of 200 µl of AgNCls/PMAA solutions were dropped onto the glass and dried under a moderate vacuum in darkness and at room temperature for 24 h, as reported in previous work (16).

*Atomic Force Microscopy:* An AFM (5,500 Scanning Probe Microscope, Agilent Technologies, Inc.) used in tapping mode was employed to characterize topographical features of the film surface. The mean resolution of this microscope was 10 nm.

### 2.2 Antibacterial effect

Agar diffusion tests (ADTs) were performed (17–19). Minimum inhibitory concentrations (MIC) (17, 19), minimum bactericidal concentration (MBC) (17, 18) and Colony forming unit/ml (CFU) (19) were determined.

Two common cariogenic bacteria, *Streptococcus mutans* and *Lactobacillus acidophilus* were used.

#### 2.2.1 Microorganism Cultivation

For *in vitro* evaluation of the inhibitory effects of the cariostatic agents, strains of *S. mutans* and *L. acidophilus* were used.

1. *S. mutans* ATCC35668 and *L. acidophilus* ATCC 9,224 were obtained from frozen stock and seeded onto Blood agar plates. The plates were incubated at 37°C for 24 h under an atmosphere of 85% N_2_, 5% CO_2_ and 10% H_2_. A single bacterial colony was then selected and used to inoculate 10 ml of enriched BHI broth (6), which was incubated at 37°C for 24 h under the same atmospheric conditions. The bacterial cells were harvested by centrifugation at 5,000 rpm, washed twice with phosphate buffered saline (pH 7.2), and their optical density was measured at 660 nm using a microplate reader. The cells were then diluted in phosphate buffered saline in 10-fold steps to a final dilution of 10^−6^.

#### 2.2.2 Determination of Minimum Inhibitory Concentration (MIC)

To evaluate the MIC of the antimicrobial agent, AgNCls/PMAA, was sequentially diluted two-fold a time from the stock solution (54 ppm of silver in AgNCls/PMAA) to a maximum dilution of 512-fold (0.1 ppm of silver in AgNCls/PMAA). The same dilution factors were used for SDF, starting at 248,400 ppm of silver and reaching a minimum concentration of 485 ppm of silver. In a 96-well plate, 100 μl of each dilution, 100 μl of bacterial culture, and 100 μl of BHI solution were added to each well. Positive controls without cariostatic agent and negative controls without cariostatic agent or microorganisms were also included.

Bacterial growth was determined by measuring the optical density (OD) of each sample at 660 nm using a spectrophotometer (SpectraMax M2/M2e Microplate Readers). The initial OD was recorded, and the plates were incubated at 37°C for 24 h in anaerobic conditions. The value for MIC was set as the minimum concentration of the antibacterial solution necessary to prevent bacterial growth after incubation.

#### 2.2.3 Determination of minimum bactericidal concentration (MBC)

Agar diffusion test (ADT) was used to calculate the minimum bactericidal concentration (MBC) of the solution. Aliquots of 10 μl were removed from the wells previously treated with antimicrobial, after 24 h of incubation at 37°C under anaerobic conditions and placed onto the Brain Heart Infusion (BHI) agar plates separately. MBC stands for the lowest concentration of the substance at which no colonies are formed.

#### 2.2.4 Colony forming unit (CFU/ml count)

Bacterial colony counting was performed using the agar plate count method. A 10 μl aliquot of diluent was extracted from the 96-well plate and dispensed onto individual Brain Heart Infusion (BHI) agar plates. Subsequently, each droplet was uniformly spread across the agar surface utilizing a sterile spatula, systematically progressing from the highest to the lowest concentration. The inoculated plates were then incubated for 48 h at 37°C. The *S. mutans* and and *L. acidophilus* CFU/ml were counted using a digital colony counter and the measurements were repeated three times.

### 2.3 Cytotoxicity

MTT assay was performed. The morphology of Dental Pulp Stem Cells (DPSCs) was observed under an inverted phase contrast microscope, and the cell viability was determined after 24 and 48 h.

#### 2.3.1 Cell culture

Commercially available dental pulp stem cells (DPSCs) were obtained and cultured in Minimum Essential Medium (MEM α) and supplemented with 10% (V/V) fetal bovine serum (FBS) and 1% (V/V) P/S (penicillin/streptomycin). The cells were then incubated at 37°C and 5% CO_2_. To ensure optimal growth, the culture medium was changed every 3–4 days until the cells reached confluence. Once the cells had reached confluence, they were detached from the culture surface using TrypLE express and passaged. For the experiment, cells from the 6th passage were utilized.

#### 2.3.2 Evaluation of cytotoxicity using MTT assay

For MTT test, MTT [3-(4,5-Dimethylthiazol-2-yl)-2,5-diphenyltetrazolium bromide] powder, dimethylsulphoxide (DMSO) and ELISA plate reader were used.

Dental Pulp Stem Cells were seeded in 96 well culture plates containing 100 μl of alphaMEM/well at a density of 7,000 cells/well and incubated for 24 h at 37°C, 5% CO_2_. The medium was then replaced with 200 µl of fresh medium containing different concentrations of AgNCls/PMAA (54, 27, 13.5, 6.75, 3.4, 1.7, 0.85, 0.42, 0.21, 0.10 ppm of silver), prepared by serial dilution. Plain culture medium was used as negative control and SDF 38% (248,400 ppm of silver) was used as a positive control. The 96 well culture plates were incubated for 24 and 48 h respectively at 37°C, 5% CO_2_. After incubation, the morphology of cells in 96 well culture plates was observed under the Inverted microscope, eclipse ti (Nikon instruments, inc). The effect of the material extracts on the cell mitochondrial function was measured by MTT assay. A 20 µl aliquot of MTT (5 mg/ml) was added into each well to a final concentration of 0.5 mg/ml, and incubated for 4 h at 37°C, 5% CO_2_. Subsequently, the wells were evacuated and 100 µl of Dimethyl Sulphoxide (DMSO) was added in darkness to each of the wells. Then, an ELISA reader quantified the optical density (OD) of the dissolved formazan crystals at 570 nm using a micro plate scanning spectrophotometer (Spectramax M2 & M2e multi-mode Microplate Reader).

The percentage of cell viability was expressed as mean ± SD according to the following formula: Viable cell % = (OD treated/OD untreated) × 100, where “OD treated” represents the absorbance value of the treated sample and “OD untreated” represents the absorbance value of the corresponding untreated sample. The cytotoxicity assay was performed in triplicates for 3 independent experiments.

### Statistical analysis

BothN for cytotoxicity and for the antibacterial effect, groups were compared using Mann–Whitney and Chi-Square tests, while intragroup comparisons at different concentrations were done using Wilcoxon and McNemar tests, with a significance set at the 95% confidence level (*p* < 0.05).

## 3 Results

### 3.1 Characterization and stability of the AgNCls/PMAA

The Ag cluster size has been characterized by Díez et al. (20). They have determined that the AgNCls/PMAA are composed of 3–5 Ag atoms with a mean clusters size smaller than 1 nm, which is below the resolution of the Optical and AFM microscopes used in this study. Therefore, only the roughness of the substrate surface was possible to observe, as reported in a previous study (16).

**Figure 1:**
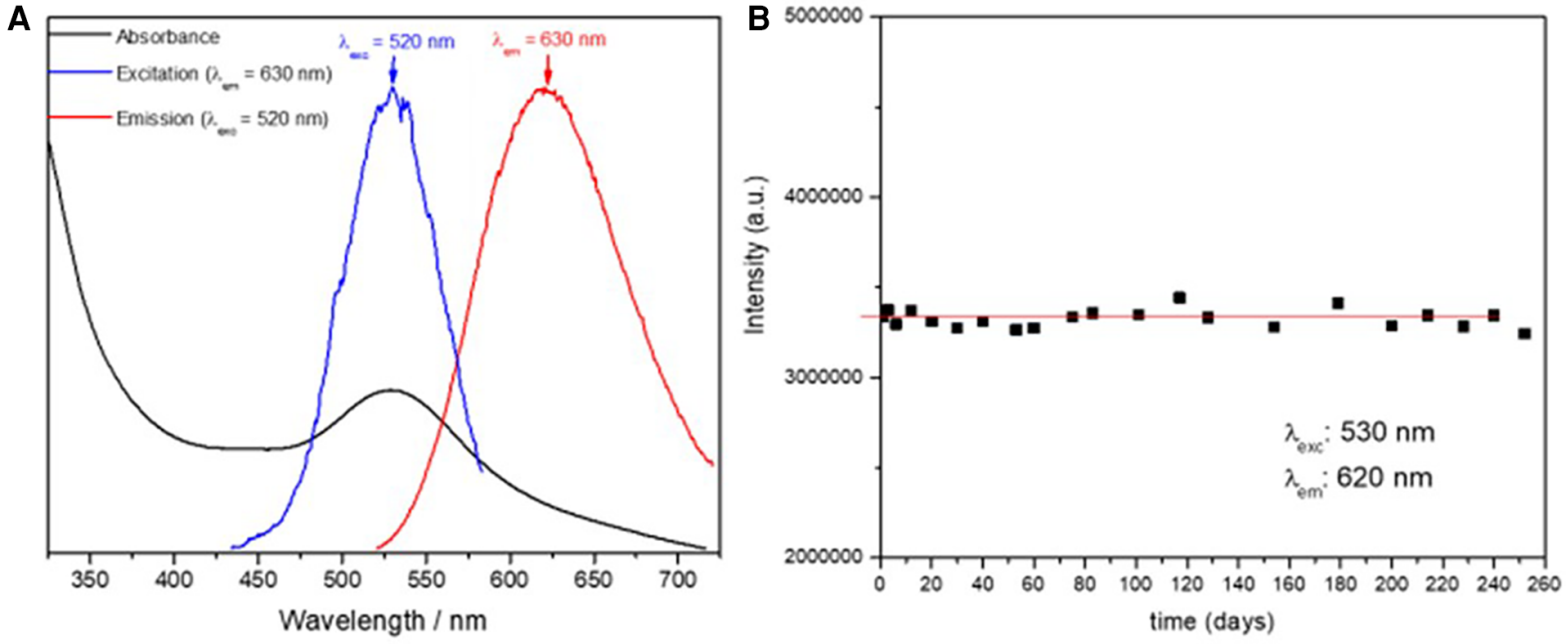
(**A**) Absorption (black), excitation recorded at λ_em _= 630 (blue) and emission recorded at λ_exc _= 520 nm, of the reference solution of AgNCIs/PMAA (54 ppm of silver). (**B**) Flourescence intensity of the reference solution of AgNCIs/PMAA (54 ppm of silver) during a 9-month period.

The UV-vis absorption spectrum as well as the excitation and emission spectra of the reference solution of AgNCls/PMAA (54 ppm of silver) were determined immediately after the clusters were synthesized and the results are shown in Figure 1A. The absorption/excitation band and the emission band are centered at 520 nm and 630 nm, respectively, as shown in Figure 1A, in agreement with the values reported by Díez et al. (20). These results strongly suggest that the size, shape and environment of the clusters prepared in this work are the same as those previously reported and that no further characterization should be needed. In addition, only a single feature centered at *λ*_exc_/*λ*_em_ = 520 nm/630 nm was observed in the EEM, suggesting that AgNCls/PMAA smaller than 1 nm and composed by 3–5 Ag atoms are the only fluorescent particles in the solution.

The stability of the reference solution of AgNCls/PMAA (54 ppm of silver) was determined throughout a 9-month period by systematically recording the EMM and the absorption spectrum periodically. The position of the absorption, excitation and emission band (λ_exc_ and λ_em_) did not show any change and no new features were observed in the EMM over 9 months, indicating that the clusters maintained their size and structure, and that no new species/particles were produced during this period.

Finally, it is desirable to avoid the presence of AgNPs since they are susceptible to oxidation and thus, to produce staining on the treated surface. The presence of AgNPs strongly quench the fluorescence of the AgNCls/PMAA solution. Therefore, the fluorescence intensity was followed up over 9 months and it remained unchanged during this period, as shown in Figure 1B, suggesting that AgNCls/PMAA are stable against coalescence and AgNPs are not formed.

These results show that the AgNCls/PMAA reference solution is stable over time and that AgNCls are well protected by the PMAA scaffold against oxidation, coalescence, agglomeration, etc. even in presence of oxygen.

### 3.2 Anti-bacterial test

#### 3.2.1 Minimum Inhibitory Concentration (MIC)

**Figure 2:**
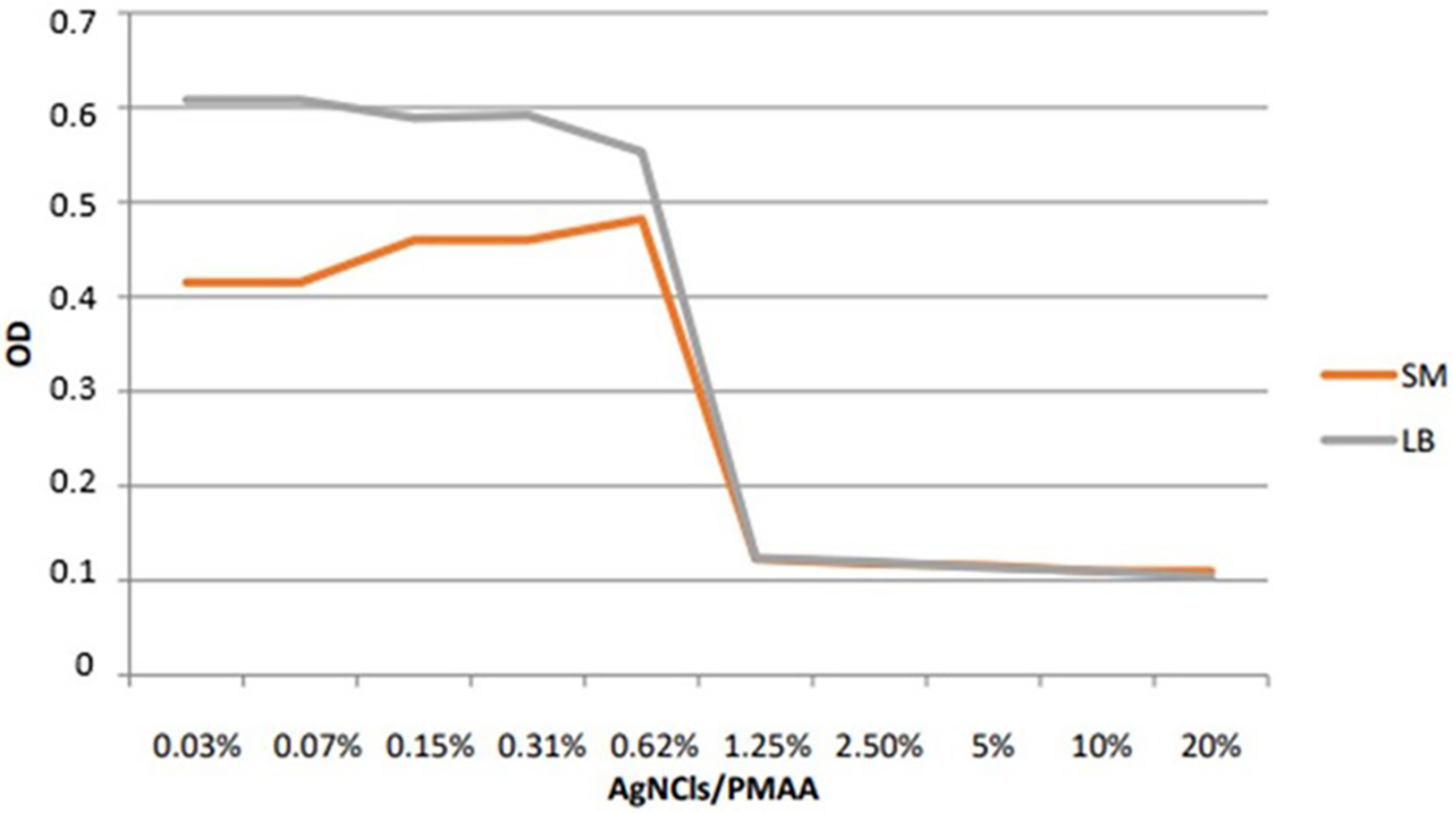
Line chart showing the optical density (OD) of *S. mutans and L. acidophilus* to different concentrations of AgNCIs/PMAA. Ref. Diluting factors 20% (1); 10% (1:2); 5% (1:4); 2.5% (1:8); 1.25% (1:16)…;0.03 (1:512).

Bacterial growth inhibition phase determined at different concentrations of AgNCls/PMAA is shown in Figure 2. In this figure, it is observed that bacterial growth inhibition phase increases with the concentration of AgNCls/PMAA until a dilution 1:16 of the reference solution, which is equivalent to 3.4 ppm of silver, where it reaches a plateau, which is extended at higher concentrations.

This very low MIC value confirms the strong antibacterial effect of AgNCls/PMAA on both bacterial strains.

#### 3.2.2 Minimum bactericidal concentration (MBC)

As shown in Table 1, the minimum bactericidal concentration (MBC) was at a 1:8 dilution for both cariogenic bacteria and was determined as the lowest concentration needed for killing the majority of the bacterial inoculums.

**Table 1:** Minimum bactericidal concentrations (MBC) of AgNCls/PMAA after 48 h.

Dilution	1:512	1:256	1:128	01:64	1:32	1:16	1:8	1:4	1:2	1
*S. mutans*	+	+	+	+	+	+	–	*–*	*–*	*–*
	+	+	+	+	+	+	*–*	*–*	*–*	*–*
	+	+	+	+	+	+	*–*	*–*	*–*	*–*
*L. acidophilus*	+	+	+	+	+	+	*–*	*–*	*–*	*–*
	+	+	+	+	+	+	*–*	*–*	*–*	*–*
	+	+	+	+	+	+	*–*	*–*	*–*	*–*

*Positive (+): Indicating growth; Negative (–): Indicating absence of growth*.

#### 3.2.3 Colony forming unit (CFU/ml count)

The CFUs of *S. mutans* and *L. acidophilus* were determined for AgNCls/PMAA at the MIC (1:16 dilution, 6.75 ppm of silver) and for SDF at the same dilution factor (1:1) for comparison. The mean CFU values obtained for both strains of bacteria are shown in Table 2.

**Table 2:** Log CFU counts of *S. mutans* and *L. acidophilus* for control (untreated) group and treated groups with AgNCls/PMAA at the MIC (1:16 dilution; 3.4 ppm of silver) and with SDF (1:16 dilution; 15,525 ppm of silver).

Treatment group	Log CFU	
	*S. mutans*	*L. acidophilus*
Control (no treatment)	13.7 ± 1.6	15.9 ± 0.8
SDF	8.6 ± 1.1	9.5 ± 0.9
AgNCls/PMAA	4.3 ± 0.4	6.3 ± 0.3
*P* value	0.01	<0.001

As observed in Table 2, the CFU/ml counts for *S. mutans* (4.3) and *L. acidophilus* (6.3) in AgNCls/PMAA (1:16, 3.4 ppm of silver) group were significantly lower than in the control groups (*p* < 0.01).

For comparison, the CFUs of both bacteria were also determined for SDF at the same dilution factor (1:16 dilution, 15,525 ppm of silver), leading to 8.6 CFU/ml and 9.5 CFU/ml, for *S. mutans* and *L. acidophilus*, respectively, as shown in Table 2.

At the same dilution of the reference solutions (1:16), AgNCls/PMAA shows to be more efficient than SDF to prevent colony formation, although the total concentration of silver in the former material (6.75 ppm) is a factor 4,600 lower than in SDF (15,525 ppm).

### 3.3 Cytotoxicity assay

**Figure 3:**
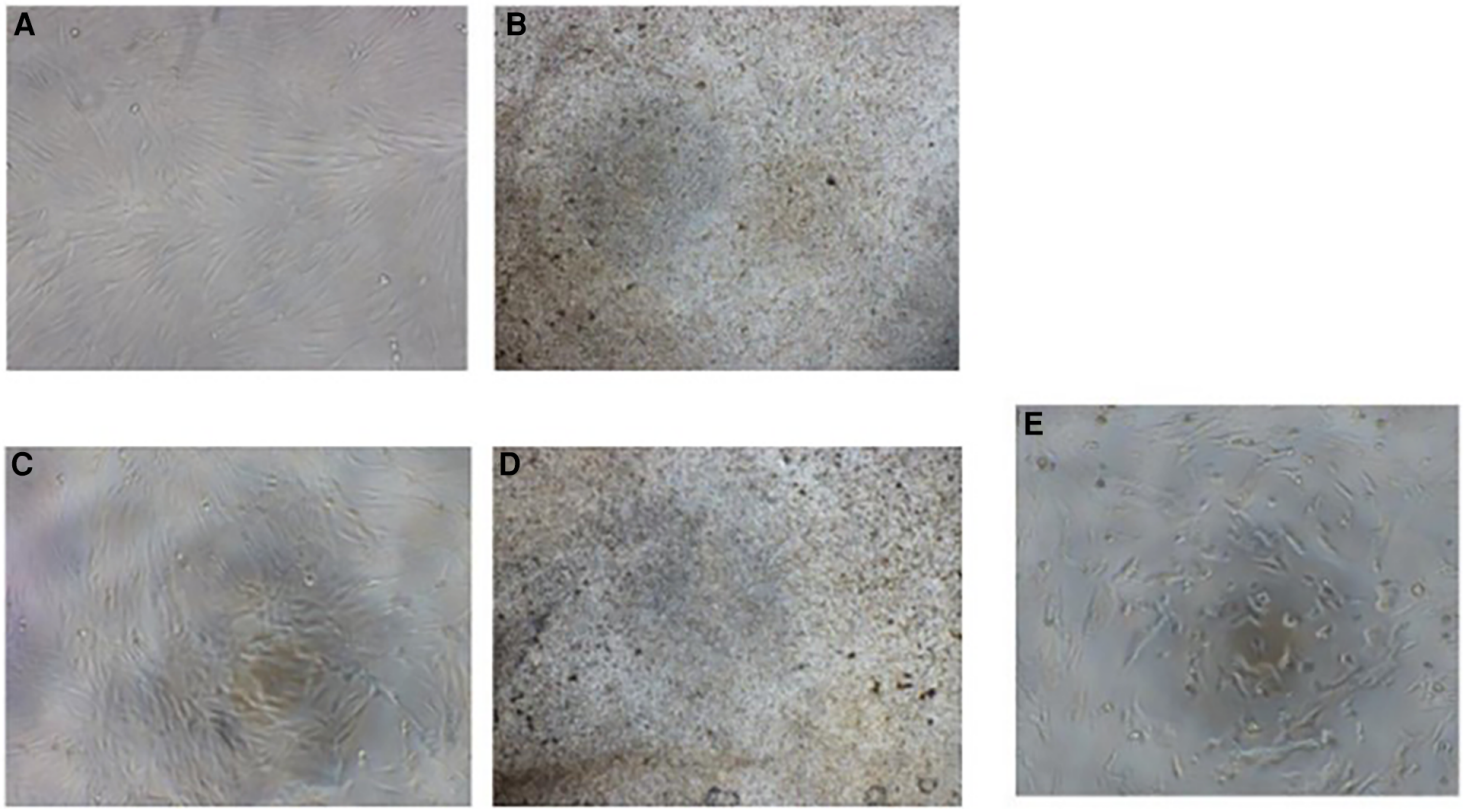
Images (**A**) and (**B**) represent DPSCs after 24 h of exposure to AgNCIs/PMAA and SDF, respectively. The images (**C**) and (**D**) show DPSCs after 48 h of exposure to AgNCIs/PMAA and SDF, respectively. Image (E) represents the negative control. Note the constant cell number and cellular morphology of DPSCs when treated with extracts of AgNCIs/PMAA.

A set of photomicrographs showing the morphology and density of cells after culturing with material extracts for 24 and 48 h is depicted in Figure 3. At the highest concentration of AgNCls/PMAA (54 ppm of silver), DPSC appeared to be fewer in number in all samples. However, normal spindle morphology was lost in cells cultured with 38% SDF (248,400 ppm of silver).

The results of cell viability experiments at different concentrations of AgNCls/PMAA and SDF after 24 and 48 h of incubation are presented in Table 3. Cell density clearly increases with decreasing concentration of the material extract.

**Table 3:** MTT results for 24 and 48-h incubation period for AgNCls/PMAA and SDF at different concentrations.

Dilution factor	Silver Concentration PPM		Mean (SD) 24 h		Mean (SD) 48 h	
	AgNC/PMAA	SDF	AgNCs/PMAA	SDF	AgNCs/PMAA	SDF
1	54	248,400	40.7 ± 0.01	19.2 ± 0.09	38.6 ± 0.05	21.4 ± 0.07
1:2	27	124,200	40.5 ± 0.01	45.3 ± 0.03	37.5 ± 0.06	40.4 ± 0.03
1:4	13.5	62,100	47.7 ± 0.07	46.3 ± 0.03	46.4 ± 0.05	39.9 ± 0.04
1:8	6.75	31,050	46.6 ± 0.05	49.3 ± 0.05	43.7 ± 0.06	43.7 ± 0.07
1:16	3.4	15,525	49.2 ± 0.06	52.9 ± 0.07	47.4 ± 0.04	44.2 ± 0.05
1:32	1.7	7,762	53.3 ± 0.08	55.5 ± 0.09	50.3 ± 0.01	49.5 ± 0.02
1:64	0.85	3,882	55.0 ± 0.03	57.8 ± 0.06	53.8 ± 0.07	52.7 ± 0.03
1:128	0.42	1,941	63.0 ± 0.05	59.6 ± 0.01	61.2 ± 0.01	58.5 ± 0.05
1:256	0.21	970	78.7 ± 0.09	67.8 ± 0.07	75.6 ± 0.05	64.4 ± 0.03
1:512	0.10	485	87.9 ± 0.01	80.0 ± 0.07	88.1 ± 0.09	78.3 ± 0.03

## 4 Discussion

To characterize this novel solution, standard tests for assessing stability, antibacterial effect and cytotoxicity were selected in order to compare the results with other studies that evaluated the same properties in similar products. SDF was used as the control group based on the many reports that demonstrated mainly its antibacterial effect derived from the presence of silver. Due to the darkening of the surfaces treated with 38% SDF (248,400 ppm of silver), caused by the oxidation of silver ions, the use of silver nanoclusters protected by different polymeric matrices have been suggested to avoid this unwanted effect (21, 22).

In this study, chemical stability of the AgNCls/PMAA composite material (54 ppm of silver) was assessed by following up the spectroscopic properties of the solutions along a 9-month period using fluorescence spectroscopy. Although it was assumed that a sustained fluorescence over time indicated a stable presence of silver, neither ion concentration nor changes in the pH were determined as in other studies on SDF.

Díez et al. (20, 23, 24) have extensively characterized the size, composition and optical properties of AgNCls/PMAA solutions. These authors determined by means of High-Resolution Mass Spectrometry (HR-MS) that AgNCls are composed of 3–5 Ag atoms, leading to a cluster size smaller than 1 nm as determined by HR-Transmission Electron Microscopy (HR-TEM). This cluster size is below the resolution of the Optical and AFM microscopes used in this study, and as reported in a previous study (16), only the roughness of the substrate surface was possible to observe. The fact that no other nanostructure was observed by AFM and optical microscopy ensures that larger nanoparticules -which are susceptible to oxidation and thus staining- are not produced or at least they are not produced at high concentration.

It has been well stablished by several authors (14, 23, 25) that these small AgNCls protected by different polymer matrices are highly fluorescent, at variance with AgNPs, and that their optical properties (absorption, excitation and emission wavelengths and fluoresce quantum yield or intensity) are strongly sensitive to the size and structure of the cluster as well as to the environment (24).

In this regard, AgNCls/PMAA smaller than 1 nm and composed of 3–5 Ag atoms have an absorption and excitation band centered at λ_exc_ = 530 nm and an emission band centered at λ_em_ = 620 nm (20, 23–25). Therefore, these optical properties can be used as a signature for the presence of the AgNCls/PMAA previously characterized by Díez et al. (20).

Silver metal is considered to have the lowest minimum inhibitory concentration (MIC) of any metal (26). Silver metal and compounds have been used as antimicrobial agents in medicine since antiquity (27) although their action mechanisms are not fully understood. It has been reported that silver causes a structural change in the bacterial cell membrane and cytoplasm. Silver ions have been shown to produce destruction of the bacterial cell wall structure, denaturation of bacterial cytoplasmic enzymes, inhibition of bacterial DNA replication, and interaction with the reactive side chain of bacterial collagenase, inactivating catalytic functions and leading to cell death (1).

Silver nanoparticles (AgNPs) have a higher surface-to-volume ratio and a smaller size to permeate cell membranes, making them unique antibacterial agents. AgNPs share a similar antibacterial mechanism mentioned above since they release silver ions in a sustained manner (28). The morphology of the silver nanoparticles, including shape and size, have a significant impact on practical application. When compared to triangular or bigger spherical silver nanoparticles, smaller spherical silver nanoparticles were shown to be superior antibacterial agents. The antimicrobial properties of these tiny silver clusters are greater than that of the large silver nanoparticles, particularly against difficult-to-treat Gram-negative pathogens (29). The results of the present study confirmed the findings of Sharma et al. (6) which showed that AgNPs presented the highest antimicrobial activity in comparison to SDF and AgNO_3_ against *S. mutans* biofilm. The antibacterial effect for AgNCls/PMAA proved to be high even at low concentrations. In this regard, it is important to highlight that the presence of silver in 38% SDF is significantly higher at all concentrations compared to any silver nanoparticle compound (1).

On the one hand, the effectiveness of AgNPs is known to be size-dependent, being inversely related to the nanoparticle size (30). On the other hand, small sizes raise concerns about biocompatibility (31). The efficacy of these small size silver particles may explain the results obtained using AgNCls/PMAA with a 1:8 dilution -a very low concentration of silver (6.75 ppm)- determined as the lowest concentration needed for killing most of the bacterial inoculums in comparison to the same dilution (1:8) but a much higher concentration of silver (31,050 ppm) for the SDF group. The high antibacterial effect on *S. mutans* and *L. acidophilus* at low concentrations of silver may also indicate that caries activity can be arrested without affecting biocompatibility.

In addition to the coverage of silver nanoparticles by different proteins or polymers, this substantial difference in the concentration of silver between SDF and AgNCls/PMAA could be addressed as another reason for reducing the staining of carious tissue as there is less likelihood of silver oxidation.

Silver nanoparticles are among the most used antibacterial agents that have been proposed to overcome the discoloration caused by the reduction of silver ions to metallic silver. Other nano antibacterial agents such as zinc oxide nanoparticles (ZnO@NP), have been introduced, which theoretically will not cause discoloration, are nontoxic, and are biocompatible which make them suitable for use in humans (32, 33). A previous investigation to this study aimed to assess the effect of ZnO@NP incorporated into resin composite for the potential one-step treatment of caries lesions. For that purpose, *Streptococcus mutans*, *Streptococcus mitis*, and *Lactobacillus acidophilus* were acquired from the Strain Collection of the Special Bacteriology Service (CCBE), INEI-ANLIS from Instituto Malbrán (Buenos Aires, Argentina). These strains were handled and kept in microaerophilic conditions using an anaerobic jar and were grown in thioglycollate broth with a colorimetric indicator or blood supplemented agar, according to the experiments needs (34).

For the present study, a similar approach was used to test the effect of silver nanoclusters on *S. mutans* and *L. acidophilus*, using a microbiologic assay with BHI agar medium for the culture of the two strains as an enriched media having dextrose, yeast extracts, and carbohydrates in their composition that facilitates their growth (6). Although an artificial monospecies cariogenic planktonic model was used in the present study, it was not able to replicate the complex oral cavity microflora; it may initially simulate biofilm changes in the presence of antimicrobial substances. Similar results to the ones presented in this study were obtained by Targino et al. (35) in an *in vitro* study to evaluate the antimicrobial activity of nanosilver fluorides in comparison to chlorohexidine and SDF.

A major limitation of this study was that the antibacterial effect was tested using planktonic assays instead of a biofilm model. Such a planktonic assay may be considered as a first step to assess the effect on silver nanoparticles on the two bacterial strains that are most commonly associated with caries (*S. mutans* and *L. acidophilus*). The widely used gold standard method to determine bacterial cell number is Colony Forming Units (CFU) counting on plates which has two main advantages, namely the capacity for counts of any number of bacteria using dilutions, if too many, or concentration is too few. Second, only viable bacteria are counted with this method as the CFU method excludes dead bacteria and debris. This method is commonly used to assess planktonic bacterial growth and susceptibility to various conditions or treatments (36). Although a biofilm model should be used as the most accurate assay to determine the antibacterial effect of silver compounds applied to arrest dentin caries lesions, a preliminary indication of the antimicrobial potential of such compounds can be suggested from planktonic assays.

Other studies carried out preliminary screening disk-diffusion susceptibility tests conducted on Mueller-Hinton agar plates inoculated with *S.mutans*, *L. acidophilus*, and *Actinomyces naeslundii* in order to identify which component of the silver compounds was responsible for any antibacterial effect (37). Information retrieved from 29 articles included after a first data collection of 1,123 publications that investigated the effect of SDF on cariogenic bacteria and dental hard tissues, eleven studies found that SDF was bactericidal to cariogenic bacteria, mainly *S. mutans* (38).

The antibacterial effect of silver nanoparticles has been reported in previous studies using microbiological assays. Elsayeda et al. (39) stated that NSF varnish had a better antibacterial effect than conventional fluoride varnish against both *S. mutans* and *S. salivarius*. One study used a combination of biofilm and planktonic assays, obtaining samples from patients and comparing with a pure strain of *S. mutans*, previously certified using ATCC: 353384 as a positive control (40). As in our study, the authors subjected SDF to monocultures and stated that the results obtained could be attributable to their use. For this reason, they suggested that research should use multispecies samples with bacteria directly associated with caries lesions. Nevertheless, the results of the present study agree with those obtained by Alvarez-Marín et al. (40) who showed the minimum concentration of 10% SDF to be effective against *S. mutans*.

The use of a planktonic assay of the two bacterial strains associated with caries activity represents a preliminary approach to study the antibacterial effect of silver nanoclusters synthesized in PMAA. However, the authors are aware that further studies using a biofilm model are needed to better understand the relation between the change in bacterial counts and the corresponding change in caries activity.

Results obtained from the MTT assay were according to expectations as it was confirmed that direct silver diammine fluoride application induced the necrosis of the pulpal cells at full concentration (41). A similar effect was observed when the pulpal cells were exposed to the AgNCls/PMAA solution, which gradually decreased with higher dilutions. Coincidentally, the concentrations with greater antibacterial effect corresponded to the ones of higher cytotoxicity. Unfortunately, same as happened with the MIC and MBC for both materials, the cytotoxicity is still high when the pulpal cells were directly exposed to the treatment solutions, which is an expected effect, since it is known that silver can cause a marked inhibition of cell growth and damage to the cell walls (42).

Interestingly, the cytotoxicity of AgNCls/PMAA and SDF was very similar for the same dilutions at 24 h, an even slightly higher for AgNCls/PMMA than for SDF in dilutions between 1:2 and 1:64, although the concentration of silver in AgNCls/PMAA was always 4 orders of magnitude lower than in SDF. This is also in line with the stronger antibacterial effect observed for the former material.

This observation may be associated to the chemical properties of PMAA that possess –COOH and –COO^−^ groups that can interact strongly and specifically with the components of the cell walls (proteins, lipids, etc.) and consequently facilitate the deposition and penetration of silver on the external membrane or in the vacuoles, serving as an adhesive material of silver. However, it is a hypothesis that needs further studies.

The fact that at higher dilutions (1:128, 1: 256 and 1:512) at 24 h the cytotoxicity of AgNCls/PMAA becomes progressively lower than the cytotoxicity of SDF under the same conditions is due to the negligible concentration of silver in AgNCls/PMAA.

Another point to highlight from the MTT assay results is that at 48 h the cytotoxicity of both materials is reverted, being SDF more cytotoxic than AgNCls/PMAA at all the dilutions, although the cytotoxicity is not significantly higher than at 24 h. This observation suggests that the effect of AgNCls/PMAA occurs very fast, immediately after application and facilitated by the adhesive capacity of PMAA. However, due to the low concentration of silver, after 24 h this material has no residual silver for a retard effect. Conversely, the silver content in SDF is considerably higher and it allows for silver to continue acting for a longer time, resulting in a higher cytotoxic effect in the long term.

A final point for discussion refers to another possible limitation of the present study regarding cytotoxicity as the tests were performed by exposing the cells directly to the treatment solutions, while indirect SDF application has shown to induce a mild inflammatory response and reparative dentin formation. It is speculated that AgNCls/PMAA cytotoxicity would be buffered by different thicknesses of dentin, preventing a negative response of pulpal cells (41).

For this study, the choice of pulpal cells attempted to simulate the challenge that may represent the application of these compounds (AgNCls/PMAA and SDF) onto the pulpal wall of a dentin caries lesion. Other studies used human gingival fibroblasts and oral squamous cell carcinoma 4 cell line (SCC 4, ATCC, VA, USA) to certify the safety and determine the selectivity index of silver nanoparticles (PEG-AgNPs). The cytotoxic effect of nanoparticles on SCC 4 and gingival fibroblasts was evaluated following the protocol of an MTT assay. Results of these studies confirmed the minimal sensitization of the gingival fibroblasts PEG-AgNPs, thus their cytological biocompatibility (43).

## 5 Conclusion

Biological properties tested for a novel AgNCls/PMAA solution to arrest dentin caries progression added valuable features to previous studies carried out to evaluate potential application of this product. The results of this work demonstrated high stability of the silver nanoclusters over time, even when exposed to oxygen, which may allow long term storage without altering its properties. The AgNCls/PMAA solution presented less cytotoxicity than SDF when pulpal cells were directly exposed to this product and a potential antibacterial effect for *S. mutans* and *L. acidophilus* at very low silver concentrations. However, biofilm model assays should be carried out to confirm its anticaries effect.

## Data Availability

The raw data supporting the conclusions of this article will be made available by the authors, without undue reservation.
